# Magnolol as a Radiotherapy Enhancer in Oral Squamous Cell Carcinoma: Targeting the EGFR/NF‐κB Pathway and Immune Modulation

**DOI:** 10.1111/jcmm.70699

**Published:** 2025-08-19

**Authors:** Yu‐Chang Liu, Chih‐Ying Liao, Fei‐Ting Hsu, Hsing‐Ju Wu, Kuan‐Tin Chen, Pei‐Hsuan Lee, Wei‐Ting Hsueh

**Affiliations:** ^1^ Department of Radiation Oncology Chang Bing Show Chwan Memorial Hospital Changhua Taiwan; ^2^ Department of Medical Imaging and Radiological Sciences Central Taiwan University of Science and Technology Taichung Taiwan; ^3^ Department of Life Sciences National Central University Taoyuan Taiwan; ^4^ Research Assistant Center, Show Chwan Memorial Hospital Changhua Taiwan; ^5^ Department of Nursing, Jenteh Junior College of Medicine Nursing and Management Miaoli Taiwan; ^6^ Department of Radiation Oncology National Yang Ming Chiao Tung University Hospital Yilan Taiwan; ^7^ Division of Radiation Oncology, Department of Oncology National Taiwan University Hospital Taipei Taiwan; ^8^ Department of Oncology National Cheng Kung University Hospital, College of Medicine, National Cheng Kung University Tainan Taiwan

**Keywords:** EGFR/NF‐κB, immunoregulation, magnolol, oral squamous cell carcinoma, radiotherapy

## Abstract

Oral cancer, particularly oral squamous cell carcinoma (OSCC), often exhibits resistance to standard treatments like surgery, chemotherapy, and radiation therapy (RT). Magnolol, a bioactive compound from the bark of Magnolia officinalis, is recognised for its anti‐inflammatory, antioxidant, antimicrobial, and antitumor properties. This study aims to explore magnolol's potential to enhance the therapeutic efficacy of RT in oral cancer models. Using MOC1‐bearing animals, we evaluated the combined effect of magnolol and RT. The results showed that magnolol significantly suppressed tumour growth, delayed progression, and reduced tumour weight compared to control groups. Immune profiling revealed that magnolol plus RT promoted positive immune regulation by increasing M1 macrophages, dendritic cells, and activated cytotoxic T cells, while suppressing negative regulators like myeloid‐derived suppressor cells (MDSCs) and regulatory T cells (Tregs). Immunohistochemical analysis also demonstrated enhanced activation of apoptosis‐related pathways including the cleavage of caspase‐3, ‐8, and ‐9. Furthermore, the combination of magnolol and RT did not induce significant toxicity, as evidenced by stable body weight, normal tissue pathology, and normal liver and kidney function markers. Notably, the phosphorylation levels of EGFR and NF‐κB were significantly reduced in the magnolol plus RT group, similar to the effects seen with erlotinib plus RT. In conclusion, these findings highlight magnolol's ability to enhance the efficacy of RT in oral cancer by targeting the EGFR/NF‐κB axis, inducing apoptosis, and modulating immune responses, presenting a promising therapeutic strategy for OSCC.

## 1 Introduction

Oral squamous cell carcinoma (OSCC) is a frequently encountered type of malignant tumour found in the head and neck area, particularly affecting the oral cavity and oropharynx [1]. The development of OSCC is closely associated with high‐risk behaviours such as smoking, betel nut chewing, and alcohol consumption, which are particularly prevalent in South and Southeast Asia [2, 3]. Surgery remains the primary treatment modality for resectable OSCC. For patients with unfavourable pathological features, postoperative radiotherapy (PORT) or concurrent chemoradiotherapy can enhance local control rates and improve survival outcomes [4, 5]. Although targeted therapy and immunotherapy are also utilised in treatment, there is an urgent need to explore and develop effective adjunctive therapies including novel pharmacological agents and alternative treatment approaches, to improve patient prognosis further [6, 7].

The mechanism by which radiation inhibits tumour cell growth through DNA damage is crucial in cancer treatment. Nevertheless, many receptor tyrosine kinases, particularly the epidermal growth factor receptor (EGFR), are often overexpressed in tumour cells. This overexpression not only promotes tumour progression but also diminishes the efficacy of radiotherapy [8, 9]. In patients with OSCC, EGFR overexpression is associated with poorer outcomes. Constitutive activation of EGFR plays a pivotal role in the development of OSCC by initiating downstream signalling pathways including PI3K/AKT/mTOR and RAF/MEK/ERK. This activation leads to several critical biological processes including cell proliferation, anti‐apoptosis, angiogenesis, and metastasis, all contributing to tumour progression and resistance to radiotherapy [10, 11]. EGFR inhibitors such as cetuximab not only inhibit the growth of OSCC but also enhance the anticancer efficacy of radiotherapy [12, 13, 14].

Traditional Chinese Medicine (TCM) is increasingly recognised as an adjunctive therapy that enhances treatment efficacy and alleviates the side effects associated with cancer therapies in patients [15, 16]. Research conducted by Ben‐Arie et al. suggests that the integration of Traditional Chinese Medicine (TCM) as an adjunctive treatment is associated with improved survival rates among patients diagnosed with oral cancer in Taiwan [2]. To comprehensively understand the therapeutic effects and underlying mechanisms of TCM in the management of oral cancer, further investigation is warranted. Magnolol is a bioactive compound extracted from the traditional Chinese medicinal plant *Magnolia officinalis*. Previous studies have reported that magnolol suppresses the activity of EGFR in triple‐negative breast cancer cells and sensitises hepatocellular carcinoma to radiation. However, it remains to be investigated whether magnolol also exhibits a radiosensitising effect in OSCC [17, 18]. Therefore, the primary objective of this study is to evaluate the therapeutic efficacy and underlying mechanisms of magnolol in combination with radiation in treating OSCC in vivo.

## 2 Material and Methods

### 2.1 Cell Culture of MOC1 Cells

The MOC1 cell line was maintained in 10 cm diameter culture dishes at 37°C in a 5% CO_2_ humidity. The MOC1 cells were cultured in IMDM (Thermo Fisher Scientific), supplemented with 5% fetal calf serum (FCS), 1% penicillin–streptomycin solution (Penn/Strep, Thermo Fisher Scientific), 5 μg/mL insulin (Sigma‐Aldrich), 40 ng/mL hydrocortisone (Sigma‐Aldrich), and 5 ng/mL epidermal growth factor (EMD Millipore) [19]. Culture‐related reagents and chemical agents were listed in Table 1.

**TABLE 1:** Reagents used in the study.

Reagents	Company	Cat no. or product no.
EGF	Sigma‐Aldrich, MO, USA	62253‐63‐8
Fetal Bovine Serum	Hyclone Laboratories Inc., Utah, UK	SH30396.02HI
Fetal calf serum	Hyclone Laboratories Inc., Utah, UK	SH30073.03
Hydrocoritsone	Sigma‐Aldrich, MO, USA	50237
Insulin	Sigma‐Aldrich, MO, USA	11070‐73‐8
IMDM	Cytiva, MA, USA	SH30003.02
Matrigel	Corning, NY, USA	356237
Penicillin/streptomycin	Cytiva, MA, USA	SV30010
Xylazine (Rompun)	Elanco, Indiana, USA	Rompun
Zoletil 50	Virbac, Carros Cedex, France	Zoletil 50

### 2.2 Establishment of MOC1 Bearing Model

Six‐ to eight‐week‐old male C57BL/6 mice were procured from the National Laboratory Animal Center (Taipei, Taiwan) and maintained in a pathogen‐free facility with access to sterilised food and water. For anaesthesia, a mixture of Zoletil 50 (50 mg/mL), xylazine (Ropum), and PBS was prepared in a volume ratio of 1:1:8. Each mouse received 100 μL of this mixture via intraperitoneal injection to induce anaesthesia. MOC1 cells were combined with Matrigel and sterilised PBS at a ratio of 3:7, resulting in a total volume of 100 μL containing 1 × 10^6^ cells, which was then subcutaneously injected into the right cheek of each mouse [20]. Tumour volume was measured bi‐daily using callipers and calculated using the formula: Volume = Height × Width^2^ × 0.523. On day 24, mice were euthanised with a double dose of the anaesthetic to ensure adequate anaesthesia, followed by cervical dislocation for tissue collection and further analysis.

### 2.3 Treatment Procedure of MOC1 Bearing Model

Upon reaching an average tumour volume of 60 mm^3^, the mice were randomly assigned to one of five experimental groups (*n* = 5 per group): the control group, the magnolol group (40 mg/kg/day), the radiotherapy (RT) group (single dose of 6 Gy on day 1), the magnolol plus RT group (40 mg/kg/day of magnolol via gavage on Day 0 and a single dose of 6 Gy on day 1), and the erlotinib plus RT group (20 mg/kg/day of erlotinib via gavage starting on Day 0 with a single dose of 6 Gy on day 1). The control group received a daily gavage of 0.1% dimethyl sulfoxide (DMSO) in 100 μL of double‐distilled water. Mice in the magnolol group were administered magnolol at a dosage of 40 mg/kg/day via gavage in 100 μL of double‐distilled water starting on Day 0. On Day 1, both the RT group and the magnolol plus RT group were subjected to a single dose of 6 Gy of radiation, delivered using a linear accelerator and specifically targeting the tumour site. Additionally, mice in the erlotinib plus RT group were treated with 20 mg/kg/day of erlotinib via gavage starting on Day 0 and received a single dose of 6 Gy of radiation on Day 1.

### 2.4 Analysis of Cytotoxicity T Cells (CTLs)

On day 24, all groups of mice were sacrificed to facilitate the isolation of spleen (SP), tumour‐infiltrating lymphocytes (TIL), and tumour‐draining lymph nodes (TDLN). The harvested tissue was processed to obtain single‐cell suspensions, which were filtered through a 40 μm strainer twice to ensure purity. Red blood cells were then lysed using ACK buffer (Thermo Fisher Scientific). After lysis, the cells were neutralised with Hank's Balanced Salt Solution buffer and subsequently stained for specific surface markers, including CD8. To detect intracellular markers such as IFN‐γ and IL‐2, the cells were fixed and permeabilized using BD fixation and permeabilization solution (BD Biosciences, Franklin Lakes, NJ, USA) according to the manufacturer's instructions. The resulting fluorescence intensity from each sample was measured with a NovoCyte flow cytometer and analysed using NovoExpress software (Agilent Technologies Inc., Santa Clara, CA, USA) [21, 22, 23]. Antibodies for flow cytometry were listed in Table 2.

**TABLE 2:** Primary antibodies used in this study for flow cytometry.

Antibodies	Company	Product no.
CD4	BD Biosciences	553051
CD8	BD Biosciences	553030
CD11b	BD Biosciences	553310
CD11c	BD Biosciences	550261
CD25	BD Biosciences	553866
CD83	BD Biosciences	742263
CD86	BD Biosciences	553692
CD206	BD Biosciences	565250
FOXP3	BD Biosciences	560403
Ly‐6G/Ly‐6C	BD Biosciences	553128
IFN‐γ	BD Biosciences	554412
I‐A/I‐E	BD Biosciences	557000
IL‐2	BD Biosciences	554429

### 2.5 Analysis of M1 Macrophage

On day 24, all groups of mice were sacrificed to isolate bone marrow (BM) and SP. For bone marrow extraction, the hind limbs of the mice were severed, and a 23G needle attached to a 3 mL syringe was used to draw HBSS (Hank's Balanced Salt Solution) for flushing out the bone marrow. The resulting cell suspension was filtered through a 40 μm strainer, followed by centrifugation. Red blood cells were lysed using ACK buffer (Thermo Fisher Scientific), and after removing the supernatant, the cells were stained with markers CD11b and CD86 for detection [24].

The spleen was ground to obtain a single‐cell suspension, filtered through a 40 μm strainer, and treated with ACK buffer to lyse red blood cells. In this case as well, the cells were stained with CD11b and CD86 markers for analysis. The fluorescence intensity from both tissues was measured using a NovoCyte flow cytometer and analysed with NovoExpress software (Agilent Technologies Inc., Santa Clara, CA, USA).

### 2.6 Analysis of M2 Macrophage

On day 24, all groups of mice were sacrificed to isolate bone BM and SP. For bone marrow collection, the hind limbs were severed, and HBSS (Hank's Balanced Salt Solution) was used to flush the marrow through a 23G needle attached to a 3 mL syringe. The obtained cell suspension was filtered through a 40 μm strainer, centrifuged, and treated with ACK buffer (Thermo Fisher Scientific) to lyse red blood cells. The cells were then stained with CD11b and CD206 markers [24].

The spleen was processed by grinding to create a single‐cell suspension, which was also filtered through a 40 μm strainer and treated with ACK buffer. Similar to the bone marrow, the spleen cells were stained with CD11b and CD206. Fluorescence intensity from both tissues was measured using a NovoCyte flow cytometer and analysed with NovoExpress software (Agilent Technologies Inc., Santa Clara, CA, USA).

### 2.7 Analysis of Dendritic Cells (DCs)

On day 24, all groups of mice were sacrificed to isolate SP, tumour‐infiltrating lymphocytes (TIL), and tumor‐draining lymph node (TDLN). The collected tissues were processed to create single‐cell suspensions, which were filtered twice through a 40 μm strainer to ensure purity. Red blood cells were lysed with ACK buffer (Thermo Fisher Scientific), and following lysis, the cells were neutralised using Hank's Balanced Salt Solution. The cells were then stained for specific surface markers, including CD11c, I‐A/I‐E, and CD83. Fluorescence intensity from each sample was subsequently measured using a NovoCyte flow cytometer and analysed with NovoExpress software (Agilent Technologies Inc., Santa Clara, CA, USA) [25].

### 2.8 Analysis of Regulatory T Cells

On day 24, all groups of mice were sacrificed to facilitate the isolation of SP, TIL, and TDLN. The harvested tissue was processed to obtain single‐cell suspensions, which were filtered through a 40 μm strainer twice to ensure purity. Red blood cells were then lysed using ACK buffer (Thermo Fisher Scientific). After lysis, the cells were neutralised with Hank's Balanced Salt Solution buffer and subsequently stained for specific surface markers, including CD4. For the detection of intracellular markers such as FOXP3 and CD25, the cells were fixed and permeabilised using BD fixation and permeabilisation solution (BD Biosciences, Franklin Lakes, NJ, USA) according to the manufacturer's instructions. The resulting fluorescence intensity from each sample was measured with a NovoCyte flow cytometer and analysed using NovoExpress software (Agilent Technologies Inc., Santa Clara, CA, USA) [25].

### 2.9 Biochemistry Analysis

After administering anaesthesia, approximately 600 μL of blood was collected from the inferior vena cava of mice on day 24. The blood was allowed to clot before being centrifuged at 1500 g for 30 min. The resulting supernatant serum was carefully harvested and stored at −80°C for later analysis. For quantification, serum samples (150–180 μL) were analysed for levels of AST, ALT, CREA, and γ‐GT using a biochemical analyser. The instrument aspirated the serum samples and directed them to different wells, where they reacted with specific reagents for each parameter. Levels of AST, ALT, CREA, and γ‐GT in each serum sample were quantified using the analytical services of Axel Biotech Inc. (Taichung, Taiwan). Statistical comparisons among the different groups were conducted using a one‐way ANOVA, facilitating a comprehensive evaluation of the observed variations [26].

### 2.10 Haematoxylin and Eosin (H&E) Staining

On day 24, following a 24‐day treatment regimen, the mice were euthanized and sacrificed. Various organs, including the heart, liver, lung, small intestine, spleen, and kidney, were collected. All organs were fixed in 10% neutral buffered formalin and stored at 4°C overnight. After fixation, the tissues were embedded in paraffin, and 5 μm‐thick sections were prepared by Bond biotech Inc. (Taichung, Taiwan). These sections were subjected to H&E staining for detailed visualisation of cellular structures, facilitating a thorough histopathological analysis [27]. Pathological images of each group were captured using the EVOS M5000 Imaging System (Invitrogen, MA, USA) at a magnification of ×100.

### 2.11 Immunohistochemistry (IHC) Staining

Resected tumours were subjected to fixation in 4% formaldehyde (PFA) at 4°C overnight, a critical step to preserve the tissue architecture and cellular details for subsequent analysis. Following fixation, the paraffin‐embedded tumour specimens were meticulously sectioned into 5‐μm thick slices by Bond Biotech Inc. (Taichung, Taiwan), ensuring uniformity and precision in the samples prepared for histological examination. To evaluate protein expression comprehensively within the tumours, IHC staining was performed, adhering rigorously to the manufacturer's instructions to guarantee reliable and reproducible results. Tumour tissues affixed to glass slides were stained with a carefully curated panel of antibodies, including anti‐EGFR (Y1068), anti‐NFκB (Ser536), anti‐cleaved Caspase 3, anti‐cleaved Caspase 8, anti‐cleaved Caspase 9, anti‐CD8, anti‐FOXP3, anti‐VEGF, anti‐IDO, anti‐CD86, and anti‐CD206, which target key proteins involved in tumour biology and immune response. The stained sections were subsequently examined using the EVOS M5000 Imaging System (Invitrogen, MA, USA) at a magnification of 100×, allowing for detailed visualisation of the immunoreactivity. For the quantitative assessment of IHC staining, Image J version 1.50 (National Institutes of Health, Bethesda, MD, USA) was utilised to analyse the intensity and distribution of the staining, providing a robust framework for data interpretation [27]. Antibodies for IHC were listed in Table 3.

**TABLE 3:** Primary antibodies used in this study for immunohistochemistry (IHC) staining.

Antibodies	Company	Product no.
CD8	ABclonal, MA, USA	A11856
CD86	Cell Signalling Technology, MA, USA	#91882S
CD206	Cell Signalling Technology, MA, USA	#24595S
EGFR (Y1068)	Cell Signalling Technology, MA, USA	#3777S
FOXP3	Cell Signalling Technology, MA, USA	#12653
IDO	Cell Signalling Technology, MA, USA	#86630S
NF‐**κ**B (Ser 536)	Cell Signalling Technology, MA, USA	#3033S
VEGF	Abcam, Cambridge, UK	ab1316
Cleaved caspase‐3	Cell Signalling Technology, MA, USA	#9661S
Cleaved caspase‐8	Cell Signalling Technology, MA, USA	#9496S
Cleaved caspase‐9	Invitrogen, MA, USA	PA5‐105271

### 2.12 Statistical Analysis

Quantitative data were expressed as mean ± standard deviation (SD) derived from three independent experiments. To evaluate statistical significance, one‐way analysis of variance (ANOVA) was conducted using GraphPad Prism version 7.0 (San Diego, CA), with a significance threshold set at *p* < 0.05. To ensure the reliability and reproducibility of the results, each experiment was performed independently at least three times, allowing for robust comparisons between the control and quetiapine‐treated groups.

## 3 Results

### 3.1 Magnolol Enhances the Treatment Efficacy of RT in Oral Cancer Models

**FIGURE 1:**
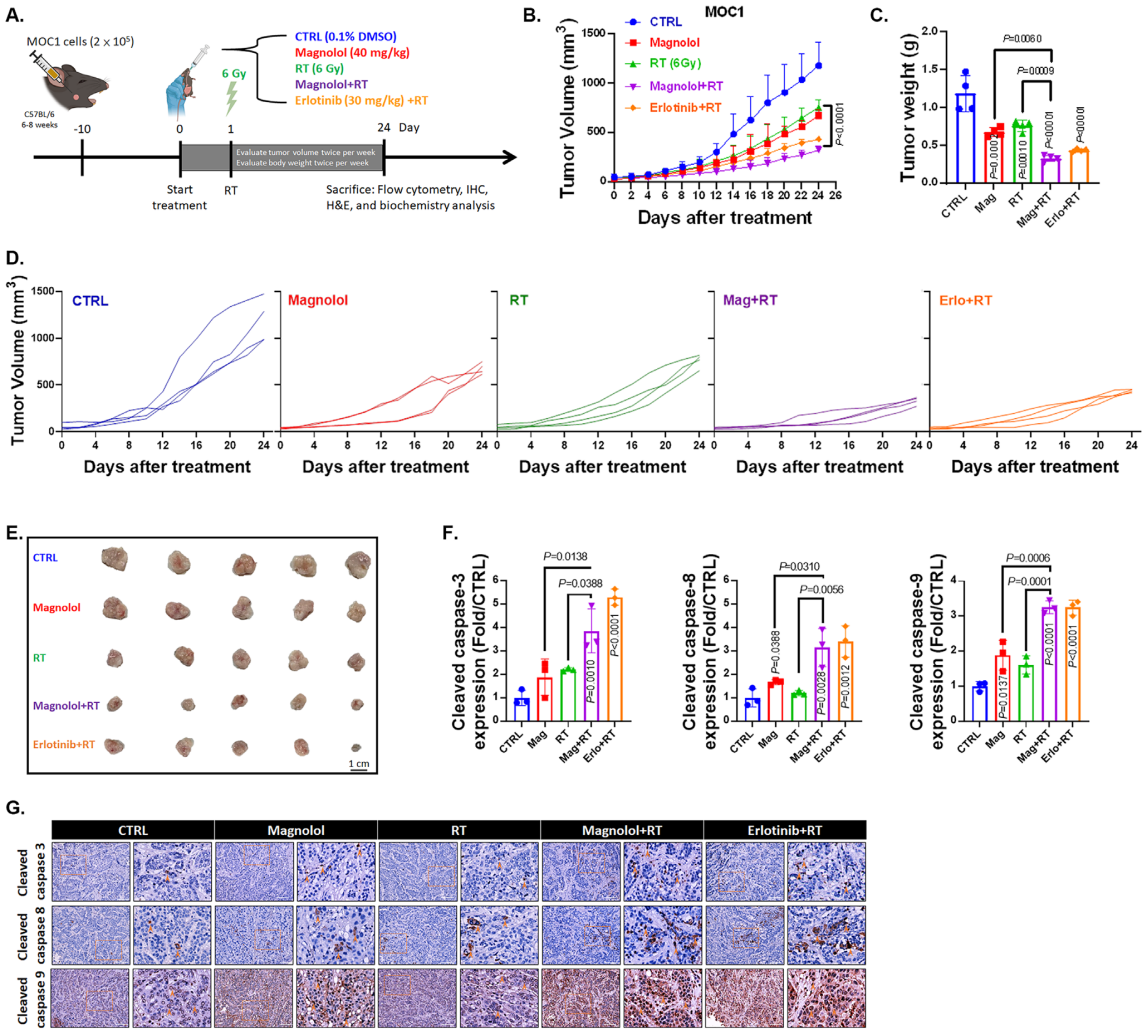
Treatment efficacy of Magnolol combined with RT on MOC1 bearing tumour. (A) Experimental flow chart for the animal study. (B) Tumour volume was measured every two days. (C) Tumour weight was recorded on day 24, and (D) tumour progression for each group of mice is shown. (E) Images of the extracted tumours are presented (*n* = 5). (F, G) The IHC staining patterns and corresponding quantification data of cleaved caspase‐3, −8, and −9 from the mouse tumours are presented.

In this study, we prepared MOC1‐bearing animals to evaluate the treatment efficacy of magnolol combined with radiation therapy (RT) (Figure 1A). Erlotinib combined with RT was used as a positive control. As shown in Figure 1B, both the magnolol plus RT and erlotinib plus RT groups demonstrated significant tumour suppression. The treatment efficacy became noticeable compared to the control group starting on day 14, compared to RT alone on day 18, and compared to magnolol alone on day 20 (Table 4). The mean time for tumour growth to reach 500 mm^3^ was 29 days in the magnolol plus RT group, which was 3.85 times slower than the control group (Table 5). Table 6 further highlights the synergistic effect of magnolol combined with RT in the MOC1 model, with a combination index of 0.65. Additionally, as shown in Figure 1C, the average tumour weight was lighter in the magnolol plus RT group compared to other treatment groups. Figure 1D displays the tumour progression in each mouse, revealing marked tumour suppression in both the magnolol plus RT and erlotinib plus RT groups. The smallest tumours were also observed in these two groups (Figure 1E). Additionally, we observed the activation of cleaved caspase‐3, ‐8, and ‐9 in tumour tissue following treatment with magnolol plus RT and erlotinib plus RT (Figure 1F,G and Table 8A). In summary, magnolol significantly enhanced the efficacy of radiation therapy in treating oral cancer and is associated with activating apoptosis‐related signalling pathways.

**TABLE 4:** Statistical analysis of tumour volume in different treatment groups and dates.

Tukey's multiple comparisons test	95% CI of diff.	Significant	Summary	Adjusted *p*
Day 0				
CTRL vs. Mag + RT	−184.2 to 216.2	No	ns	0.9995
Mag vs. Mag + RT	−199.7 to 200.8	No	ns	> 0.9999
RT vs. Mag + RT	−189.4 to 211.0	No	ns	0.9999
Day 14				
CTRL vs. Mag + RT	153.1 to 553.5	Yes	****	< 0.0001
Mag vs. Mag + RT	−102.4 to 298.0	No	ns	0.6636
RT vs. Mag + RT	−66.08 to 334.4	No	ns	0.3511
Day 16				
CTRL vs. Mag + RT	270.4 to 670.8	Yes	****	< 0.0001
Mag vs. Mag + RT	−45.69 to 354.8	No	ns	0.2136
RT vs. Mag + RT	−17.65 to 382.8	No	ns	0.0924
Day 18				
CTRL vs. Mag + RT	412.9 to 813.3	Yes	****	< 0.0001
Mag vs. Mag + RT	−1.387 to 399.1	No	ns	0.0526
RT vs. Mag + RT	46.55 to 447.0	Yes	**	0.0074
Day 20				
CTRL vs. Mag + RT	470.9 to 871.3	Yes	****	< 0.0001
Mag vs. Mag + RT	47.71 to 448.1	Yes	**	0.0070
RT vs. Mag + RT	99.34 to 499.8	Yes	***	0.0005
Day 22				
CTRL vs. Mag + RT	564.3 to 964.8	Yes	****	< 0.0001
Mag vs. Mag + RT	86.71 to 487.1	Yes	**	0.0010
RT vs. Mag + RT	176.0 to 576.5	Yes	****	< 0.0001
Day 24				
CTRL vs. Mag + RT	652.3 to 1053	Yes	****	< 0.0001
Mag vs. Mag + RT	146.1 to 546.5	Yes	****	< 0.0001
RT vs. Mag + RT	228.1 to 628.6	Yes	****	< 0.0001

*Note:* Significance Value indicates *p < 0.05; **p < 0.01; ***p < 0.0005 and ****p < 0.0001.

**TABLE 5:** The mean tumour growth time, delay time, and inhibition rate in MOC1 tumour‐bearing mice after treatment under different conditions are presented.

Treatment	MTGT (day)^a^	MTGDT (day)^b^	MGIR^c^
CTRL	10.18	N.A.	N.A.
Magnolol	17.99	7.81	1.77
RT	16.17	5.99	1.59
Mag + RT	39.18	29.00	3.85

Abbreviation: N.A., Not available.

^a^ Mean tumour growth time (MTGT): The expected timeframe when the MOC1 tumour volume reaches 500 mm^3^.

^b^ Mean tumour growth delay time (MTGDT): The disparity between the MTGT of the treated group and that of the CTRL group.

^c^ Mean growth inhibition rate (MGIR): The MTGT of the treated group divided by the mean tumour growth time of the CTRL group.

**TABLE 6:** The mean tumour growth inhibition rate and combination index in MOC1 tumour‐bearing mice after treatment with magnolol, radiotherapy (RT), and Mag + RT are presented.

Xenografts	Magnolol	RT	Mag + RT		Index^d^
	MGIR^a^	MGIR^a^	Expected^b^	Observed^c^	
MOC1	0.43	0.36	0.57	0.72	0.65

^a^ Mean growth inhibitory rate (MGIR): 1‐(the 24th day's mean tumour volume ratio of the treated group/the 24th day's mean tumour volume ratio of the control group).

^b^ Expected growth inhibitory rate: growth inhibition rate of Magnolol × growth inhibition rate of RT.

^c^ Observed growth inhibitory rate: growth inhibition rate of Mag + RT.

^d^ Index value was calculated by (1 − MGI of combination)/(1 − Expected growth inhibitory rate). An index < 1.0 indicates a synergistic effect.

### 3.2 Magnolol Combined With RT Does Not Appear to Induce General Toxicity in Oral Cancer Models

**FIGURE 2:**
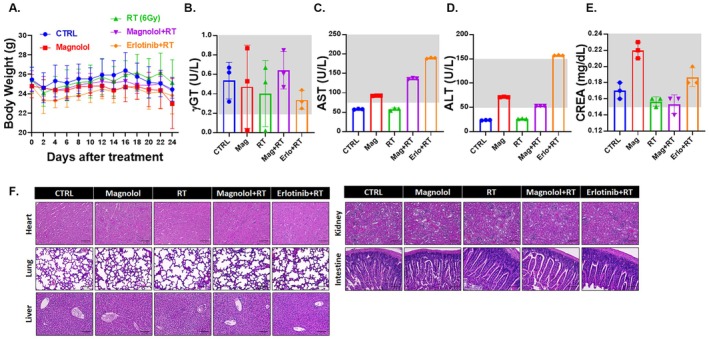
No apparent general toxicity was observed in Magnolol combined with RT treatment. (A) Body weight was recorded every two days across all treatment groups. Serum levels of (B) γGT, (C) AST, and (D) ALT, representing liver function, are shown. (E) Serum levels of CREA, representing kidney function, are also presented. Gray area represented as the normal level of different factors. (F) H&E staining on various organs after different treatments are presented.

As shown in Figure 2A, there were no significant changes in body weight across all treatment groups during the entire treatment period. This stability in body weight suggests that the treatments did not induce adverse effects typically associated with toxicity, such as weight loss or anorexia, which are common indicators of general systemic distress in animal models. We further evaluated liver function by assessing key markers, including gamma‐glutamyl transferase (γGT), aspartate aminotransferase (AST), and alanine aminotransferase (ALT). Figure 2B–D illustrate that the levels of γGT, AST, and ALT remained within the normal physiological range in both the magnolol plus radiation therapy (RT) and erlotinib plus RT groups. The maintenance of these liver function markers indicates that neither treatment adversely affected hepatic function, which is critical for overall health and metabolic processes. Additionally, we measured creatinine (CREA) levels to assess kidney function. As shown in Figure 2E, CREA levels were within the normal range across all treatment groups, confirming that the therapies administered did not compromise kidney function. To comprehensively evaluate potential organ toxicity, we conducted histopathological analyses using haematoxylin and eosin (H&E) staining on various organs, including the heart, lungs, liver, spleen, kidneys, and intestines. The analysis revealed no observable pathological changes in any of the treatment groups (Figure 2F and Table 7), indicating that the magnolol plus RT treatment did not result in significant damage or dysfunction of these vital organs. These findings suggest that the magnolol combined with RT treatment does not exhibit significant general toxicity, highlighting its potential as a safe therapeutic option in the context of oral cancer treatment.

**TABLE 7:** Severity scores for pathological alterations after different treatments.

Group	Organ type	Score of the region			
		R1	R2	R3	R4
CTRL #1	Heart	0–1	0–1	0–1	0–1
CTRL #2		1	0	0–1	0–1
Magnolol #1	Heart	0–1	0	0	0
Magnolol #2		0–1	0	0–1	1
RT #1	Heart	0	1	1–2	2
RT #2		0–1	0–1	2	2
Mag + RT #1	Heart	0	0	0	0–1
Mag + RT #2		0	0	0	0
CTRL #1	Liver	1	0	0–1	0–1
CTRL #2		0–1	1	0–1	1
Magnolol #1	Liver	0	0–1	0	0
Magnolol #2		0	1	0	0–1
RT #1	Liver	1	0–1	2	0–1
RT #2		2	0–1	0–1	2
Mag + RT #1	Liver	0	0	0	0–1
Mag + RT #2		0–1	0	0	0–1
CTRL #1	Kidney	2	2	0–1	1
CTRL #2		0	0–1	1	0–1
Magnolol #1	Kidney	0–1	0	0	0
Magnolol #2		0–1	0	0–1	0
RT #1	Kidney	0	0	0–1	0
RT #2		0–1	0	2	0
Mag + RT #1	Kidney	0	0	0	0–1
Mag + RT #2		0–1	0	0–1	0
CTRL #1	Spleen	1	0–1	0	0
CTRL #2		0	0–1	0–1	0
Magnolol #1	Spleen	0	0	0	0–1
Magnolol #2		0–1	0	0	0
RT #1	Spleen	0	0–1	0–1	0
RT #2		0–1	2	0–1	0–1
Mag + RT #1	Spleen	0	0	0–1	0
Mag + RT #2		0–1	0	0	0
CTRL #1	Small intestine	0	0	0	0–1
CTRL #2		0	1	0–1	1
Magnolol #1	Small intestine	0	0–1	0	0
Magnolol #2		0	0	0	0
RT #1	Small intestine	2	2	0	0
RT #2		1	2	2	2
Mag + RT #1	Small intestine	0–1	0	0	0
Mag + RT #2		0	0	0–1	0

*Note:* The alteration of pathology after different treatments were assessed using the following four severity scores: 0—regular tissue, 1—mild changes, 2—moderate changes, 3—significant changes. The interpretation of the pathological findings was conducted by a veterinarian with over 5 years of experience in pathological interpretation. Each group of mice was examined using two different slices (Slice #1 and Slice #2), with 3–4 regions analysed per slice.

### 3.3 Magnolol Combined With RT Promotes Positive Immune Regulation in Oral Cancer Models

**FIGURE 3:**
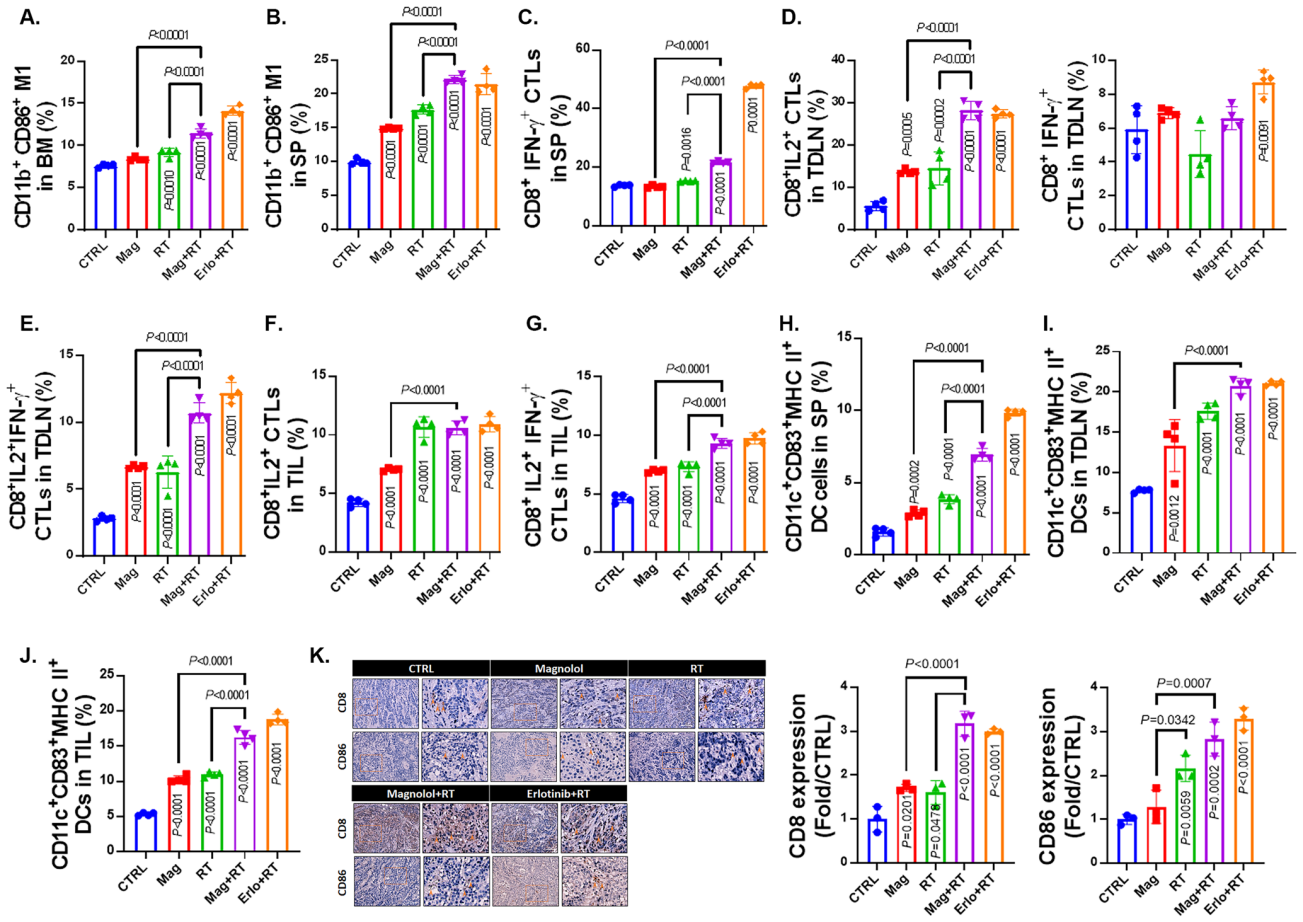
Promoted positive immune regulation was observed in Magnolol combined with RT treatment. (A) M1 macrophages (CD11b^+^/CD86^+^) in bone marrow (BM) and (B) spleen (SP) were analysed by flow cytometry. CTLs (CD8^+^/IFN‐γ^+^) in SP and (C–E) CTLs (CD8^+^/IL‐2^+^, CD8^+^/IFN‐γ^+^, and CD8^+^/IL‐2^+^/IFN‐γ^+^) in tumour‐draining lymph nodes (TDLN) were observed using flow cytometry. (F, G) CTLs (CD8^+^/IL‐2^+^ and CD8^+^/IL‐2^+^/IFN‐γ^+^) in tumour‐infiltrating lymphocytes (TIL) were also assessed by flow cytometry. (H, I, J) CD11c^+^/CD83^+^/MHC‐II^+^ dendritic cells (DCs) in SP, TDLN, and TIL were measured using flow cytometry. (K) The IHC staining patterns and corresponding quantification data of CD86 and CD8 from the mouse tumours are presented.

As shown in Figure 3A,B, an accumulation of M1 macrophages (CD11b^+^/CD86^+^) was observed in the bone marrow and spleen of the magnolol plus RT treatment group. M1 macrophages are known for their pro‐inflammatory and tumoricidal properties, which are crucial in initiating anti‐tumour immune responses [28]. The enhanced presence of these cells in the magnolol plus RT group suggests that the treatment promotes an immune‐supportive microenvironment, potentially aiding in the suppression of tumour growth. Additionally, there was an increase in activated cytotoxic T cells (CTLs), including CD8^+^/IFN‐γ^+^, CD8^+^/IL‐2^+^, and CD8^+^/IL‐2^+^/IFN‐γ^+^ cells, in the spleen and tumour‐draining lymph nodes (Figure 3C–E). Notably, a higher accumulation of CD8^+^/IL‐2^+^ and CD8^+^/IL‐2^+^/IFN‐γ^+^ CTLs was observed in the magnolol plus RT group compared to other groups (Figure 3F,G). Elevating these activated CTLs in the magnolol plus RT group indicates that the combination treatment enhances immune surveillance and activates these key immune cells, which are critical for effective anti‐tumour responses [29]. To further assess whether magnolol plus RT promotes antigen presentation, we evaluated dendritic cells (DCs). As shown in Figure 3H–J, CD11c^+^/CD83^+^/MHC‐II^+^ DCs in the spleen, tumour‐draining lymph nodes, and tumour‐infiltrating lymphocytes were 1.5–2 times more abundant in the magnolol plus RT group compared to the control group. This increase in DCs suggests that magnolol combined with RT may enhance tumour antigen presentation to T cells, thereby amplifying the anti‐tumour immune response [30]. The protein levels of CD86 and CD8 in the tumour, as assessed by IHC, were significantly increased in the magnolol plus RT group (Figure 3K and Table 8B). In summary, magnolol combined with RT effectively boosted key immune regulatory factors across multiple immune compartments. The treatment promoted the accumulation of M1 macrophages, which support anti‐tumour immunity, increased the activation and proliferation of cytotoxic T cells, and enhanced the presence of dendritic cells responsible for antigen presentation. These findings collectively indicate that magnolol in combination with RT strengthens the immune system's ability to recognise and attack tumour cells, offering a promising therapeutic strategy for oral cancer.

**TABLE 8:** (A) Statistical analysis of apoptosis‐related proteins in different treated groups from IHC is presented in Figure 1G. (B) Statistical analysis of immunostimulatory‐related proteins in different treated groups from IHC is presented in Figure 3K. (C) Statistical analysis of immunosuppressive‐related proteins in different treated groups from IHC is presented in Figure 4E.

Tukey's multiple comparisons test	95% CI of diff.	Significant	Summary	Adjusted *p*
(A) Apoptosis‐related				
Cleaved caspase‐3				
CTRL vs. Mag + RT	−4.438 to −1.289	Yes	***	0.0010
Mag vs. Mag + RT	−3.548 to −0.3996	Yes	*	0.0138
RT vs. Mag + RT	−3.227 to −0.07809	Yes	*	0.0388
Cleaved caspase‐8				
CTRL vs. Mag + RT	−3.520 to −0.7990	Yes	**	0.0028
Mag vs. Mag + RT	−2.848 to −0.1274	Yes	*	0.0310
RT vs. Mag + RT	−3.313 to −0.5927	Yes	**	0.0056
Cleaved caspase‐9				
CTRL vs. Mag + RT	−2.963 to −1.549	Yes	****	< 0.0001
Mag vs. Mag + RT	−2.076 to −0.6608	Yes	***	0.0006
RT vs. Mag + RT	−2.355 to −0.9406	Yes	***	0.0001
(B) Immunostimulant‐related				
CD8				
CTRL vs. Mag + RT	−2.772 to −1.569	Yes	****	< 0.0001
Mag vs. Mag + RT	−2.063 to −0.8595	Yes	****	< 0.0001
RT vs. Mag + RT	−2.165 to −0.9620	Yes	****	< 0.0001
CD86				
CTRL vs. Mag + RT	−2.647 to −1.013	Yes	***	0.0002
Mag vs. Mag + RT	−2.359 to −0.7245	Yes	***	0.0007
RT vs. Mag + RT	−1.481 to 0.1533	No	ns	0.1288
(C) Immunosuppressive‐related				
FOXP3				
CTRL vs. Mag + RT	0.4663 to 0.8242	Yes	****	< 0.0001
Mag vs. Mag + RT	0.1226 to 0.4805	Yes	**	0.0018
RT vs. Mag + RT	−0.03971 to 0.3182	No	ns	0.1522
CD206				
CTRL vs. Mag + RT	0.04054 to 0.8838	Yes	*	0.0306
Mag vs. Mag + RT	−0.2132 to 0.6300	No	ns	0.5144
RT vs. Mag + RT	−0.2580 to 0.5853	No	ns	0.7098
IDO				
CTRL vs. Mag + RT	0.1111 to 0.4300	Yes	**	0.0017
Mag vs. Mag + RT	−0.09348 to 0.2255	No	ns	0.6626
RT vs. Mag + RT	0.08743 to 0.4064	Yes	**	0.0033
VEGF				
CTRL vs. Mag + RT	0.2964 to 0.8657	Yes	***	0.0004
Mag vs. Mag + RT	−0.04569 to 0.5237	No	ns	0.1126
RT vs. Mag + RT	0.5485 to 1.118	Yes	****	< 0.0001

*Note:* Significance Value indicates *p < 0.05; **p < 0.01; ***p < 0.0005 and ****p < 0.0001.

### 3.4 Magnolol Combined With RT Suppressed Negative Immune Regulation in Oral Cancer Models

**FIGURE 4:**
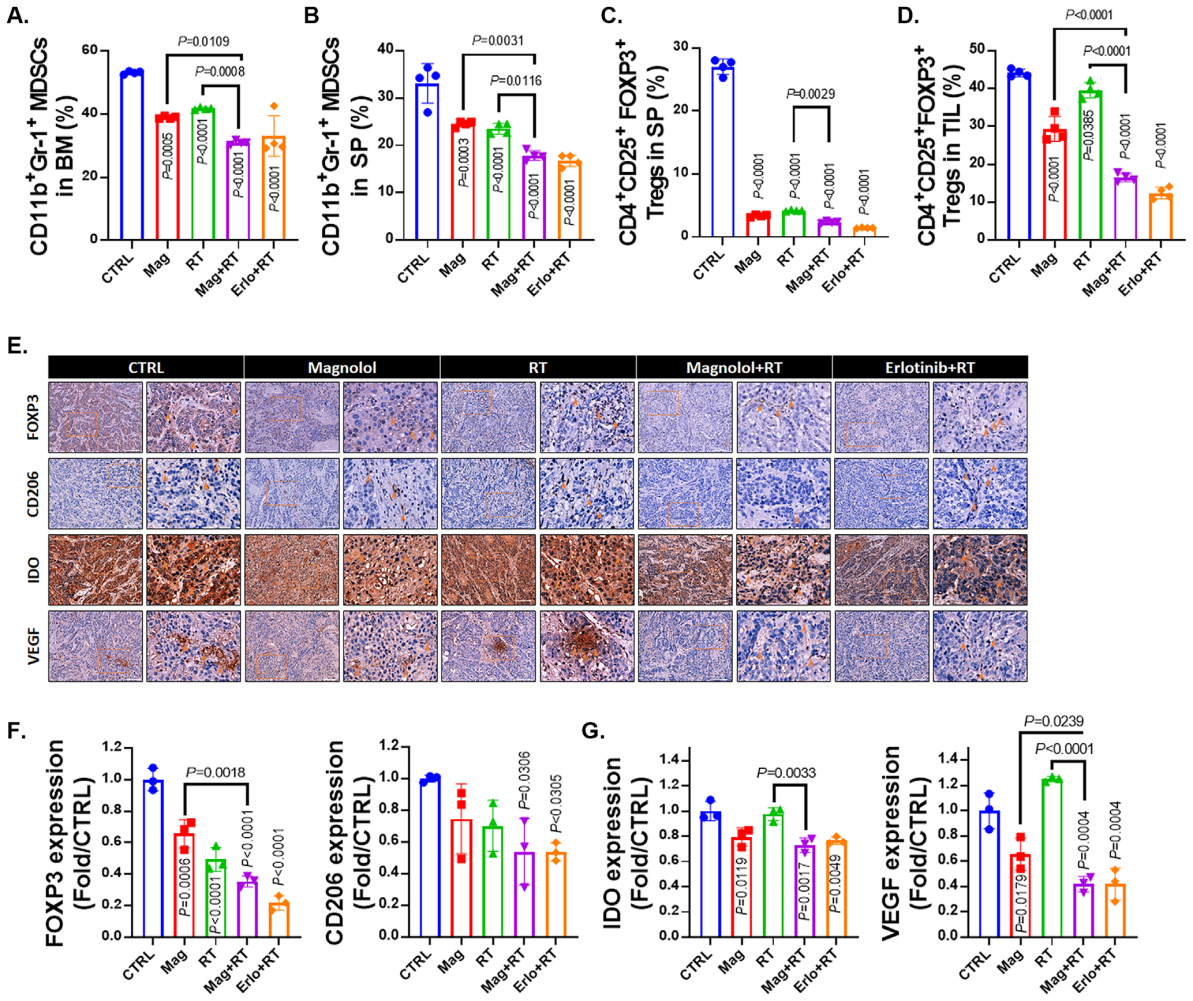
Suppressed negative immune regulation was detected in Magnolol combined with RT treatment. (A) MDSCs (CD11b^+^/Gr‐1^+^) in BM and SP were analysed by flow cytometry. (C, D) Tregs (CD4^+^/CD25^+^/FOXP3^+^) in SP and TIL were measured using flow cytometry. (E–G) The IHC staining patterns and corresponding quantification data of FOXP3, CD206, IDO and VEGF from the mouse tumours are presented.

After confirming the positive immune regulatory effects of magnolol combined with RT, we also investigated its impact on negative immune regulatory factors, which are known to suppress the immune system's ability to target tumours. First, we observed that magnolol plus RT led to a reduction in myeloid‐derived suppressor cells (MDSCs) in both the bone marrow (BM) and spleen (SP) (Figure 4A,B). MDSCs are immunosuppressive cells that inhibit the activation of T cells and promote tumour progression by creating a suppressive tumour microenvironment [31]. The reduction in MDSCs in the magnolol plus RT group suggests that the combination treatment not only enhances immune activation but also diminishes the presence of these suppressive cells, thereby potentially improving the immune system's ability to fight the tumour. Additionally, we noted a significant decrease in regulatory T cells (Tregs) characterised by the markers CD4^+^/CD25^+^/FOXP3^+^ in the SP and TIL (Figure 4C,D). Tregs are another key player in immune suppression, as they dampen the immune response and protect the tumour from immune attack [32]. The reduction of Tregs in the magnolol plus RT group implies a shift towards a more favourable immune environment, where immune suppression is reduced, allowing for a more robust anti‐tumour response. Additionally, the protein level of FOXP3, an important transcription factor for Tregs, was decreased in the tumour as assessed by IHC (Figure 4E,F and Table 8C). The tumour‐associated macrophage marker CD206 was also decreased by magnolol plus RT. Furthermore, the immunosuppressive factors VEGF and IDO were reduced in tumour tissue with the magnolol combined with RT treatment (Figure 4E,G and Table 8C). In summary, magnolol combined with RT promotes positive immune regulation by increasing the activity of cytotoxic T cells, macrophages, and dendritic cells but also inhibits negative immune regulation by reducing the populations of immunosuppressive MDSCs and Tregs. These dual effects suggest that magnolol plus RT may effectively reprogram the tumour microenvironment to favour immune‐mediated tumour destruction, enhancing the therapeutic potential of this combination treatment in oral cancer.

### 3.5 Magnolol Combined With RT Inhibited OSCC Progression Is Associated With Inactivation of EGFR/NF‐κB Axis

**FIGURE 5:**
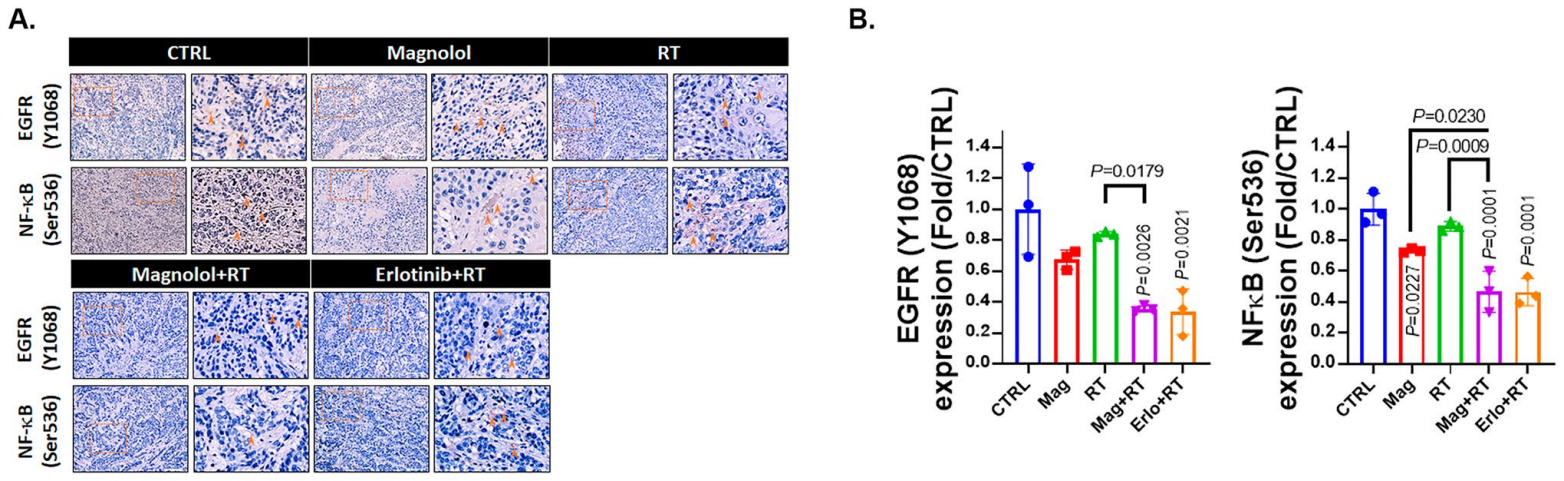
Inactivation of EGFR/NF‐κB axis was observed in Magnolol combined with RT treatment. (A, B) The IHC staining patterns and corresponding quantification data of EGFR (Y1068) and NF‐κB (Ser536) from the mouse tumours are presented.

To further investigate the regulatory mechanisms of magnolol in oral squamous cell carcinoma (OSCC), we performed immunohistochemical (IHC) staining on tumour tissue samples to assess key molecular changes. One of the primary objectives was to determine whether the anti‐OSCC effects of magnolol mirror those of erlotinib, particularly in relation to the inactivation of epidermal growth factor receptor (EGFR) signalling. As shown in Figure 5A–B, magnolol combined with radiation therapy (RT) significantly decreased the phosphorylation of EGFR, a key marker of its activation, similar to the effects observed with erlotinib combined with RT treatment. This inhibition was substantial, with magnolol plus RT reducing the levels of phosphorylated EGFR (P‐EGFR) by more than half compared to the control group. In addition to EGFR, we also evaluated the downstream signalling pathways to understand the broader impact of magnolol. Specifically, we examined the phosphorylation of nuclear factor kappa B (NF‐κB), a well‐known transcription factor involved in inflammation and cancer progression, which is activated downstream of EGFR [33]. The results indicated that the phosphorylation of NF‐κB was also significantly reduced in the magnolol plus RT group, paralleling the reduction observed in the erlotinib plus RT group. This suggests that magnolol's anti‐tumour activity is associated with the suppression of the EGFR/NF‐κB signalling axis, which plays a critical role in OSCC progression.

## 4 Discussion

Previous study has demonstrated that magnolol inhibits the growth of OSCC by enhancing antitumour immunity [16]. In this investigation, we further explored the radiosensitising effects and underlying mechanisms of magnolol on OSCC in vivo. The results revealed that tumour volume in the combination group was significantly reduced compared to the groups treated with radiation or magnolol alone (Figure 1B–E). Apoptosis is a critical mechanism through which anticancer drugs and radiation eliminate tumour cells, primarily by activating both extrinsic and intrinsic caspase‐mediated pathways. In this process, caspases‐8 and ‐9 act as initiators in the extrinsic and intrinsic pathways, respectively, while caspases‐3, ‐6, and ‐7 function as effector caspases [34, 35, 36]. The results demonstrated a significant increase in the expression of cleaved caspase‐3, ‐8, and ‐9 in the combination treatment group compared to the groups treated with either radiation or magnolol alone (Figure 1F,G). Magnolol enhances radiation‐induced apoptosis through both extrinsic and intrinsic pathways. These findings suggest that magnolol can sensitise OSCC cells to radiation.

Most cancer patients undergo radiotherapy during treatment, primarily aimed at controlling the growth of local tumours and helping to eliminate distant metastatic cancer cells. Previous research largely focused on the direct destruction of cancer cells by radiotherapy, but scientists have gradually discovered that the immune system also plays a crucial role in radiotherapy, particularly in radiotherapy‐induced anti‐tumour immune responses, where the immune system can further assist in tumour regression [37, 38]. A previous study has reported that magnolol enhances anti‐tumour immunity in an orthotopic MOC1‐bearing model. Building on these findings, the present study further examines the effects of magnolol on radiotherapy‐induced anti‐tumour immune responses in vivo, specifically within an OSCC model.

CTLs, DCs, and M1 macrophages have crucial roles and are essential for the anti‐tumour immune response induced by radiation [37]. Through death ligands or the perforin/granzyme B pathways, activated CTLs effectively induce apoptosis in tumour cells. DCs simultaneously initiate and promote the anti‐tumour functions of specific T cells through effective antigen presentation and costimulatory signals. M1 macrophages kill tumour cells by releasing cytotoxic molecules or utilising antibody‐dependent cell‐mediated cytotoxicity (ADCC) [39, 40, 41]. Based on flow cytometry analysis, we found that radiation promoted the accumulation of activated CTLs, DCs, and M1 macrophages in the spleen, TDLN, TIL, and BM, with this response effectively enhanced by treatment with magnolol (Figure 3A–J). Furthermore, according to IHC staining results, we observed a significant increase in the expression of CD86 (an M1 macrophage marker) and CD8 (a CTL marker) in the tumour tissue of the combination treatment group (Figure 3K). A high density of tumour‐infiltrating CD8^+^ T cells has been reported to correlate with favourable outcomes in OSCC [42].

Immunosuppressive cells and molecules play pivotal roles in shaping the tumour microenvironment (TME) and facilitating tumour immune evasion. Among these, myeloid‐derived suppressor cells (MDSCs) and regulatory T cells (Tregs) are prominent immunosuppressive cell types within the TME. These cells exert their effects primarily by inhibiting the activity of CTLs and DCs, both of which are critical for eliciting effective anti‐tumour immune responses. IDO and VEGF, immunosuppressive molecules expressed by tumour and immunosuppressive cells, not only suppress the proliferation and maturation of CTLs and DCs but also promote the expansion of MDSCs and Tregs, further amplifying the immunosuppressive effect within the tumour microenvironment [43, 44, 45]. Our data demonstrate that magnolol effectively enhances the radiation‐induced reduction of both MDSCs and Tregs in the spleen, BM, and TILs (Figure 4A–D). Furthermore, the expression of VEGF and IDO is significantly reduced in the combination group compared to the radiation alone group (Figure 4E,G).

EGFR is a receptor tyrosine kinase that drives tumour progression and upregulates the TME by activating downstream oncogenic kinases and transcription factors. Within the TME, EGFR inhibits anti‐tumour immune cells and recruits' immunosuppressive cells through inducing the expression of cytokines, chemokines, and other effector proteins. NF‐κB, as an oncogenic transcription factor, plays a significant role in the control of tumour progression and the microenvironment by EGFR. In the TME, hyperactivation of the EGFR/NF‐κB pathway increases tumour cell resistance to radiation, thereby reducing the efficacy of radiotherapy. This pathway also promotes the expression of immunosuppressive proteins such as IDO and VEGF, which suppress antitumor immune cells and promote the accumulation of immunosuppressive cells. These effects impair antitumor immunity and contribute to tumour growth [46, 47, 48, 49, 50]. In the results, erlotinib was found to not only effectively enhance radiation‐induced tumour growth suppression, apoptosis, and the levels of anti‐tumour immune cells but also strengthen the inhibitory effects of radiation on immunosuppressive cells and proteins (Figures 1, 3 and 4). Additionally, IHC staining analysis revealed that magnolol effectively enhances the radiation‐induced decrease in EGFR and NF‐κB activity in OSCC tissues (Figure 5). Magnolol may exert this effect by downregulating EGFR activity, thereby suppressing downstream NF‐κB signalling. Inactivation of this pathway has been reported to enhance both radiosensitivity and antitumor immunity [14, 47, 49]. Consistently, we observed that magnolol not only promotes radiation‐induced apoptosis but also amplifies radiation‐induced antitumor immune responses, both of which contribute to the suppression of OSCC growth.

In conclusion, our study provides compelling evidence for the in vivo radiosensitizing efficacy of magnolol on OSCC growth. As a potential radiosensitizer, magnolol not only enhances radiation‐induced tumour growth inhibition, apoptosis, and the levels of anti‐tumour immune cells, including CTLs, M1 macrophages, and DCs, but also highlights the inhibitory effects of radiation on immunosuppressive cells and proteins, such as Tregs, MDSCs, IDO, and VEGF. Our data suggest that the inactivation of EGFR/NF‐κB signalling is associated with the enhanced inhibitory efficacy of magnolol in combination with radiation in OSCC in vivo.

## 5 Limitation

Before progressing to clinical application, magnolol requires comprehensive preclinical evaluation, including dose optimization, chronic toxicity studies, and detailed pharmacokinetic profiling. Additional challenges include the potential for interactions with existing therapies, regulatory approval complexities, and the need for reliable and efficient drug delivery methods. Despite these limitations, magnolol remains a promising therapeutic candidate with strong potential for integration into future cancer treatment strategies, provided these barriers are systematically addressed.

## Author Contributions

**Yu‐Chang Liu:** conceptualization (lead), data curation (lead), writing – original draft (lead). **Chih‐Ying Liao:** data curation (lead), writing – original draft (equal). **Fei‐Ting Hsu:** conceptualization (equal), data curation (equal), writing – original draft (supporting), writing – review and editing (equal). **Hsing‐Ju Wu:** data curation (equal), formal analysis (equal), writing – original draft (supporting). **Kuan‐Tin Chen:** funding acquisition (equal), investigation (equal), writing – original draft (supporting). **Pei‐Hsuan Lee:** funding acquisition (equal), writing – original draft (supporting). **Wei‐Ting Hsueh:** validation (equal), visualization (equal), writing – original draft (lead), writing – review and editing (lead).

## Conflicts of Interest

The authors declare no conflicts of interest.

## Supporting information


**Appendix S1:** jcmm70699‐sup‐0001‐AppendixS1.docx.

## Data Availability

The data supporting the findings of this study are available from the corresponding author upon reasonable request.
