# Supplementary material for: Magnolol as a Radiotherapy Enhancer in Oral Squamous Cell Carcinoma: Targeting the EGFR/NF‐κB Pathway and Immune Modulation
Source: J Cell Mol Med. 2025 Aug 19;29(16):e70699. doi: 10.1111/jcmm.70699 (PMC12364617; doi:10.1111/jcmm.70699)
Supplement: Supplementary file 1 — Appendix S1: jcmm70699‐sup‐0001‐AppendixS1.docx. [file JCMM-29-e70699-s001.docx]

**Supplementary figures**

**
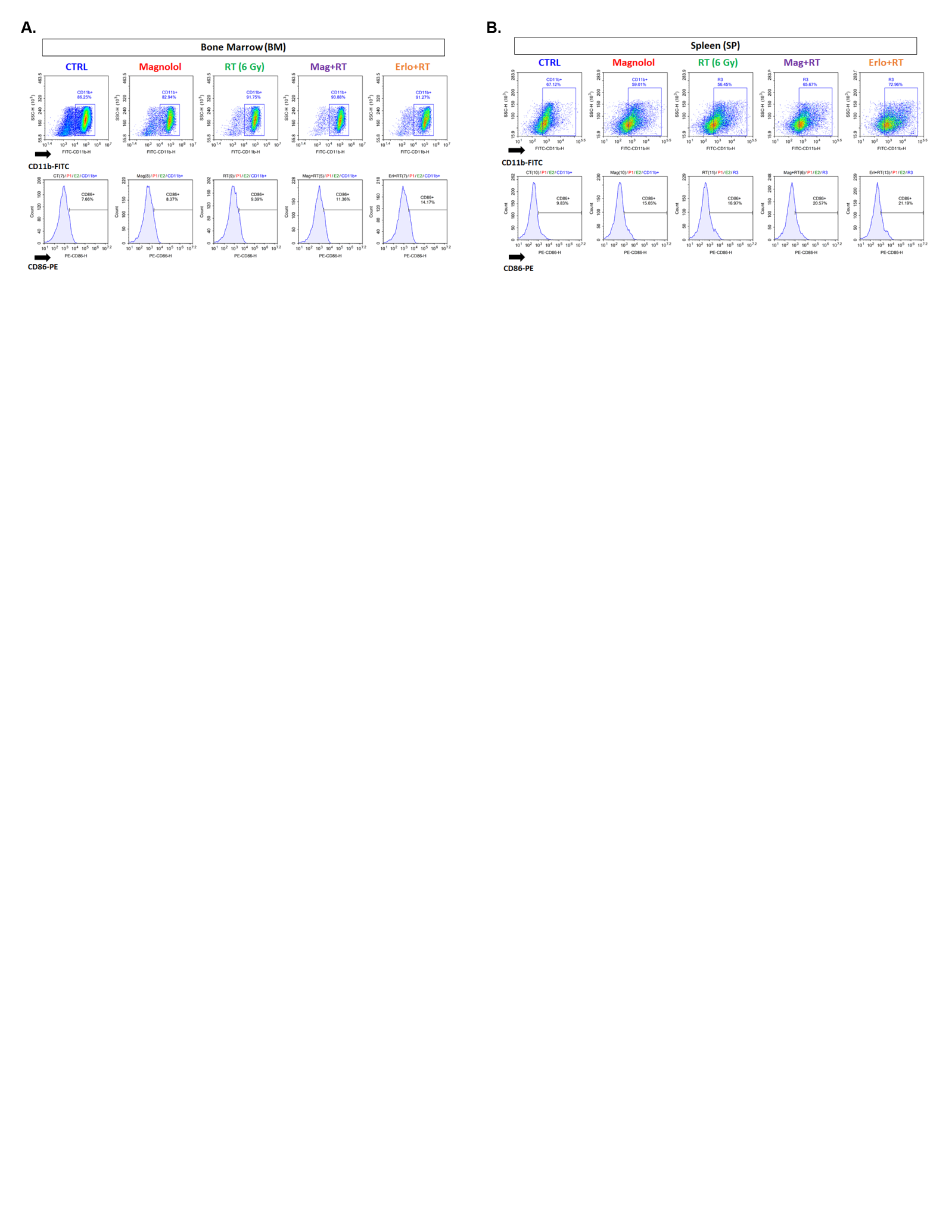
**

**Supplementary figure 1.** Flow cytometry expression pattern of M1 macrophage from (A) bone marrow and (B) spleen presented in figure 3A-B.

**
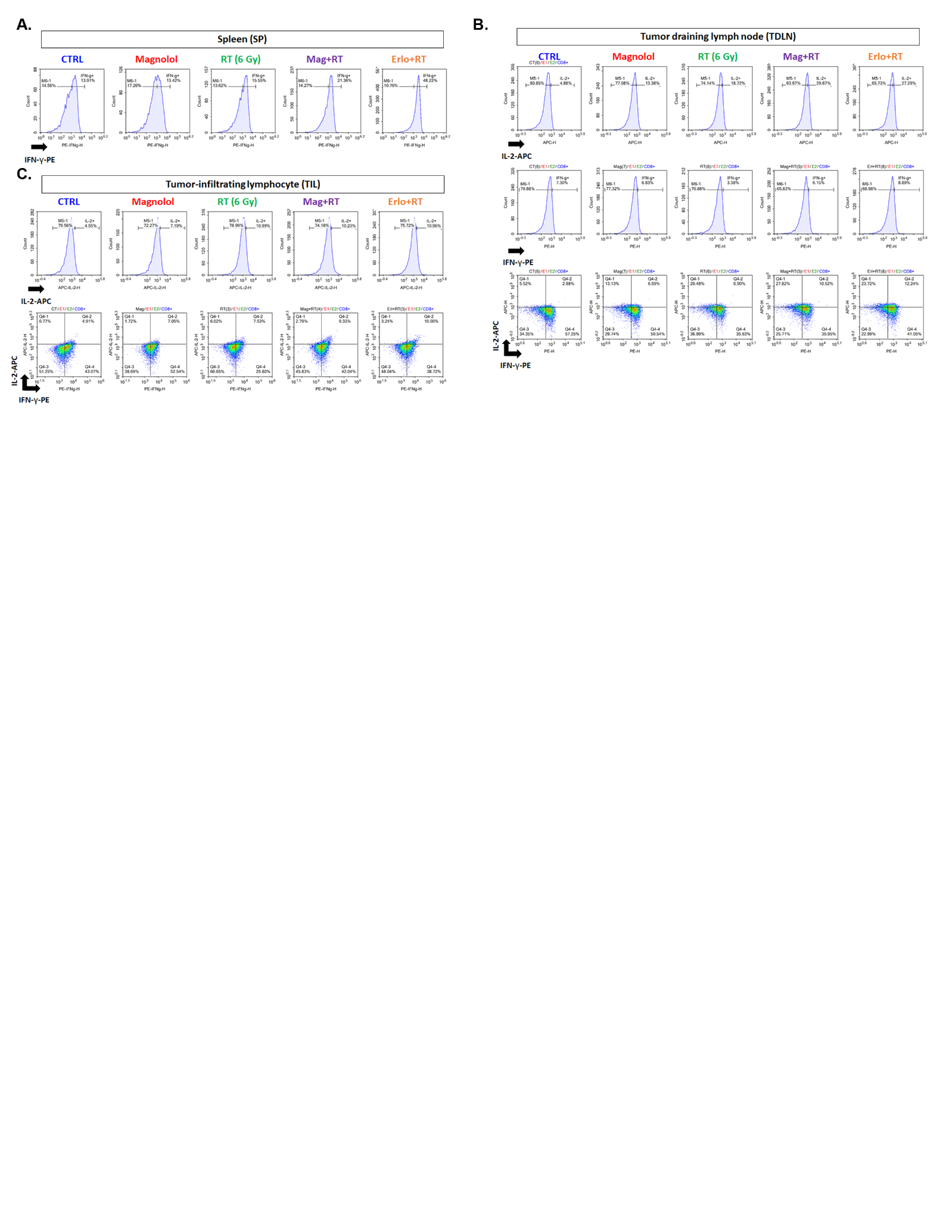
**

**Supplementary figure 2.** Flow cytometry expression pattern of CD8^+^IFN- γ ^+^, CD8^+^IL-2^+^ or CD8^+^ IL-2^+^IFN-γ^+^ CTLs from (A) spleen, (B) TDLN and (C) TIL presented in figure 3C-G.

**
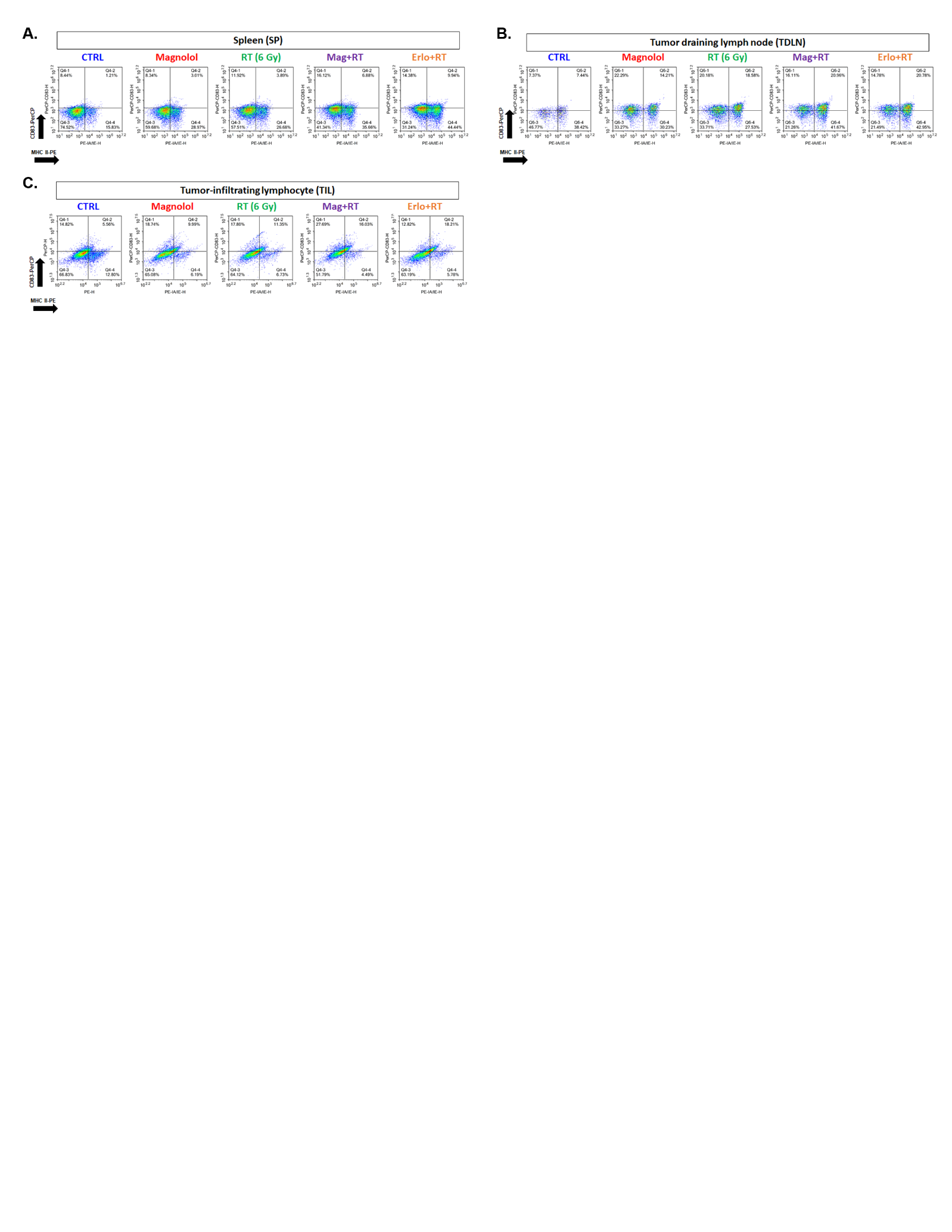
**

**Supplementary figure 3.** Flow cytometry expression pattern of CD11c^+^CD83^+^MHCII^+^ DCs from (A) spleen, (B) TDLN and (C) TIL presented in figure 3H-J.

**
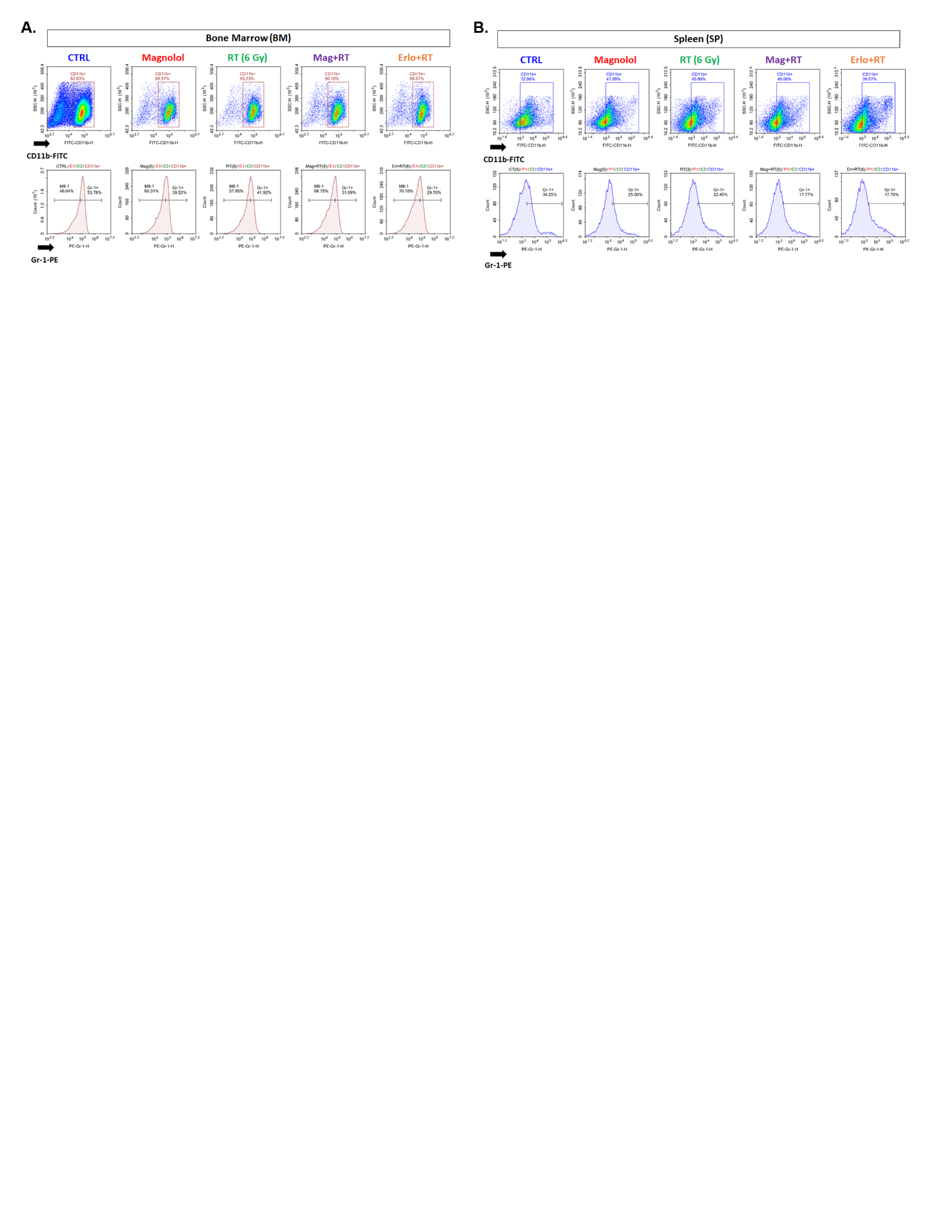
**

**Supplementary figure 4.** Flow cytometry expression pattern of CD11b^+^Gr-1^+^ MDSCs from (A) bone marrow, and (B) spleen in figure 4A-B.

**
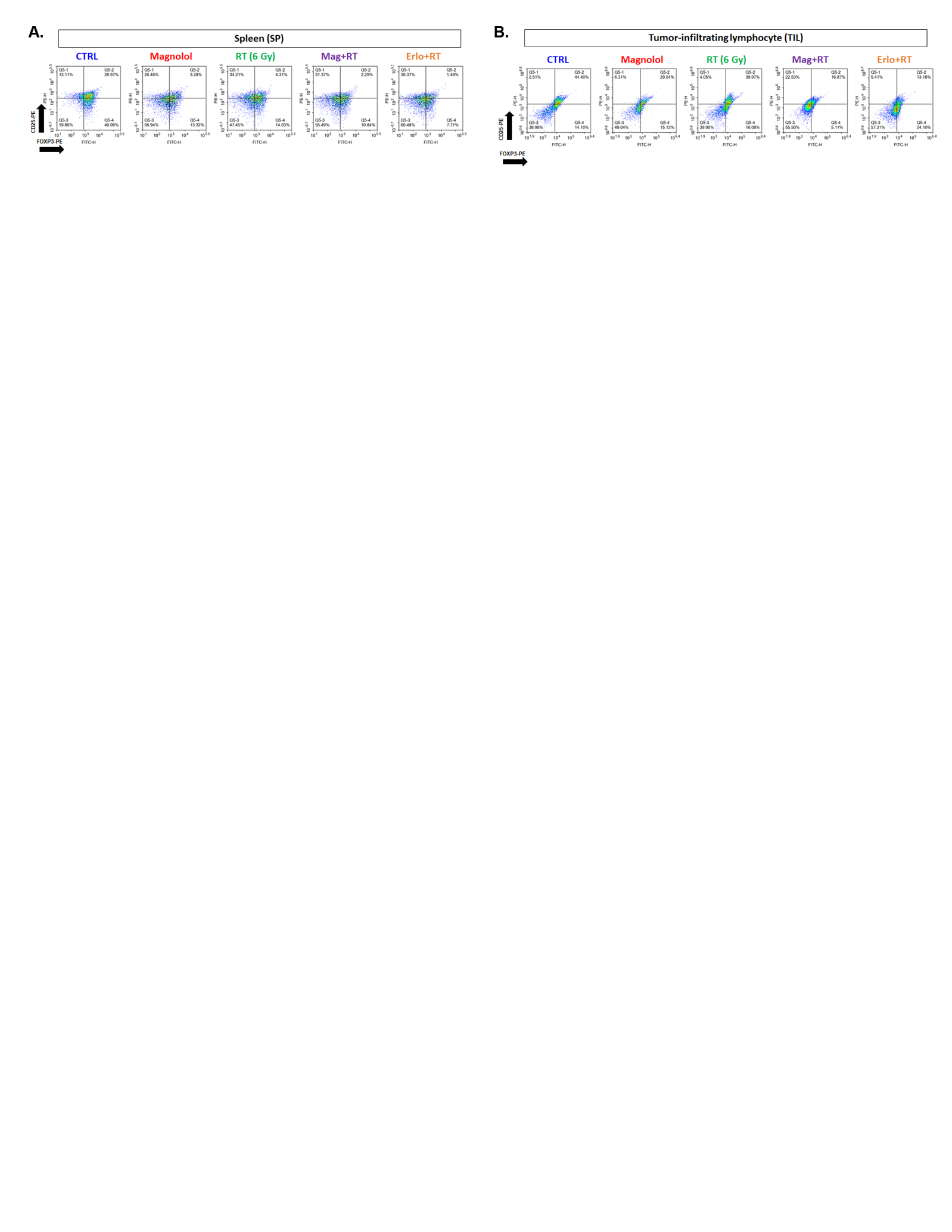
**

**Supplementary figure 5. Supplementary figure 4.** Flow cytometry expression pattern of CD4^+^CD25^+^ FOXP3^+^ Tregs from (A) spleen and (B) TIL in figure 4C-D.
